# Women’s health and healthcare experiences in the years after gestational diabetes or hypertensive disorders of pregnancy

**DOI:** 10.1186/s12884-025-07296-7

**Published:** 2025-02-14

**Authors:** Bec Jenkinson, Valerie Charlton, Leah Hardiman, Ayme Limmer, Melanie McKenzie, Anna-Lee Ura, Carissa Bonner, Sheleigh Lawler, Philippa Middleton, Gita Mishra, Jenny Doust

**Affiliations:** 1 School of Public Health, The University of Queensland, Brisbane, Australia; 2 Australian Action on Pre-Eclampsia, Central Coast, Australia; 3 Consumer Representative, Brisbane, Australia; 4 Harrison’s Little Wings, Brisbane, Australia; 5 Australasian Birth Trama Association, Gold Coast, Australia; 6 School of Public Health, University of Sydney, Sydney, Australia; 7 South Australian Health and Medical Research Institute, Adelaide, Australia; 8 University of Adelaide, Adelaide, Australia

**Keywords:** Diabetes, Gestational, Hypertension, Pregnancy-induced, Cardiovascular diseases, Preventive health services, Focus groups, Qualitative research

## Abstract

**Background:**

Pregnancy complications, such as gestational diabetes mellitus (GDM) and hypertensive disorders of pregnancy (HDP), affect a significant proportion of women in Australia, with long-term implications for cardiovascular disease (CVD) risk. Despite existing preventive measures, participation in ongoing health monitoring remains low. This study aims to explore women’s preferences and experiences regarding preventive healthcare after GDM and HDP, and to identify their unanswered questions about the association between these conditions and future CVD risk.

**Methods:**

A participatory, qualitative approach was adopted, involving a Lived Experience Expert Group (LEE Group) to plan, conduct, and interpret focus groups with women who had experienced either GDM or HDP. Participants were recruited through health consumer and community organisations and took part in two focus groups conducted via Zoom. The focus groups involved a stimulus presentation about CVD and GDM or HDP, facilitated group discussion about participants’ health and healthcare since their pregnancy, and Nominal Group Technique to prioritise participants’ questions about their CVD risk. Focus groups were audio recorded and transcripts from each group were analysed thematically. Synthesised Member Checking was used to verify the trustworthiness of findings.

**Results:**

Twelve women participated in the focus groups, with distinct themes emerging from the GDM and HDP focus groups. Participants were previously unaware of the association between their pregnancy complication and increased risk of future CVD and wished to know more. Three themes were generated from the GDM focus groups: ‘*a distressing diagnosis*’; ‘*degrees of diabetes*’; and ‘*balancing motherhood and self-care*’. Two themes were generated from the HDP focus groups: ‘*women’s concerns were dismissed*’ and ‘*wanting follow up at the right time and with the right person*’. The ‘top ten’ questions from each group focussed on improving maternity care, preventing CVD, and (for the HDP group) concerns beyond CVD.

**Conclusions:**

Women’s capacity to engage in preventive health after GDM and HDP is influenced by their maternity care experiences and the accessibility of primary care pathways. Future interventions should focus on improving woman-centred maternity care, ensuring seamless transitions to primary care, and addressing the social determinants of health for new mothers.

**Supplementary Information:**

The online version contains supplementary material available at 10.1186/s12884-025-07296-7.

## Background

Pregnancy complications, such as hypertensive disorders of pregnancy (HDP) and gestational diabetes (GDM), affect about 6% and 19% [1] (respectively) of women who give birth in Australia each year. Beyond their immediate impact on perinatal outcomes, these conditions are also associated with other long-term health issues. Women who had either of these conditions are up to twice as likely to develop cardiovascular disease (CVD) than other women [2, 3].

Adding female specific risk factors, such as a history of GDM or HDP, to contemporary CVD risk calculators for women aged 45–69 does not appear to improve the ability to identify the women most at risk of CVD [4]. However, because GDM and HDP occur several years or even decades before the development of CVD, there is an opportunity to institute or continue preventive healthcare after pregnancy to reduce this risk [5]. This is especially important since symptoms of cardiometabolic disease may be evident within five years of these pregnancy complications [6, 7], but may be dismissed or misdiagnosed, particularly in younger women [8].

Interventions to improve monitoring and health behaviour change in women after GDM, aiming to improve diet, physical activity, or both, have shown mixed results [9–12]. Since 2011, Australia has had a National Gestational Diabetes Register that aims to improve risk awareness, promote diabetes prevention, and encourage regular diabetes screening. However rates of participation in ongoing screening for Type 2 Diabetes Mellitus (T2DM) after GDM remain low [13, 14], with barriers to uptake identified as time and caring responsibilities [15].

In the case of HDP, only two randomised controlled trials have been conducted to test lifestyle interventions in women 6–12 months post-partum [16, 17]. The lifestyle interventions in both studies involved coaching regarding healthy eating and physical activity. Reports arising from these studies acknowledge difficulties with sustaining change in lifestyle health behaviours beyond the intervention period, with a major barrier being lack of time [16]. Further pilot studies of lifestyle interventions after HDP [18, 19] have been undertaken, and definitive studies planned [20], but for both GDM and HDP, participant retention [11] and long term behaviour change [16] have proved challenging [21]. Although clinical guidelines recommend ongoing monitoring after HDP [22, 23], uptake is low [14].

Questions remain about what information women want about CVD risk after GDM or HDP, when they want to receive it, or what advice they should be given about how to reduce their risk of CVD [24, 25]. This knowledge is needed to inform the co-design of future preventive health interventions for these women. Therefore, this study aimed to describe women’s preferences and experiences of preventive healthcare in the years after GDM and HDP, and to identify their unanswered questions about the association between their pregnancy complication and future CVD risk.

## Methods

### Study design

This study adopted a participatory, qualitative approach, collaborating with a Lived Experience Expert Group (LEE Group) to plan, conduct and interpret a series of focus groups, with women who had previously experienced GDM or HDP. Members of the LEE group (*n* = 6) were drawn from Australian maternity consumer organisations, such as Australian Action on Pre-eclampsia, Preterm Infants Parents Association, and the Australasian Birth Trauma Association. In the absence of a specific Australian health consumer organisation focussed on GDM, an individual consumer advocate interested in GDM was invited. This paper is reported in line with the Guidance for Reporting Involvement of Patients and the Public (GRIPP2) [26] and Consolidated Criteria for Reporting Qualitative Research [27].

### Reflexivity and researcher characteristics

Reflexivity allows researchers to identify the background, values and assumptions that they bring to the research process, increasing rigour and transparency. Our author team is multidisciplinary, and includes members of the LEE group, current health professionals, and public health researchers. In addition to their own lived experience of GDM or HDP, some of the LEE Group authors are also leaders of Australian maternity consumer and peer support organisations. The first author (BJ) is a maternity consumer advocate, who undertook doctoral study and is now an early career researcher. Throughout this study, BJ engaged in regular individual reflection and group discussion, to surface assumptions, and test emerging ideas and decisions with the multidisciplinary team.

### Participants and sampling

Recruitment for this study was undertaken in collaboration with relevant health consumer organisations, such as Australian Action on Pre-Eclampsia and Australasian Birth Trauma Association, other community groups, such as the Ethnic Communities Council of Queensland, and through online support groups, such as “Gestational Diabetes Australian Support” on Facebook. Each network was invited to nominate eligible people to take part in the study. Formally constituted organisations were offered a $200 donation to acknowledge their assistance with recruitment, and were provided with social media assets, including a 90 s animated explainer video (see Supplementary Material). This approach was intended to ensure that participants were connected to peer support networks, able to represent the views and experiences of others and more likely to be comfortable and experienced discussing potentially distressing topics. People were eligible to participate in the study where they were living in Australia, at least 18 years old, had experienced either GDM or HDP at least one year ago, and not currently pregnant. Participants also needed to be willing to participate in online focus group conducted in English. In keeping with the Consumers Health Forum of Australia’s definition of a consumer representative [28], registered health care professionals were ineligible to participate in the study.

As nominations were received from collaborating consumer and community organisations, a member of the research team contacted interested people to discuss the study and answer any questions, before seeking written informed consent to participate. During this initial conversation, the researcher discussed any support that the participant might need to take part, including offering pre-focus group digital familiarisation sessions. We anticipated that about 12 participants would be needed to deliver the necessary information power to achieve the study aims [29]. Information power [29] is a qualitative approach to anticipating sample size, which requires a subjective but informed assessment. In our study, this assessment considered the narrow focus of the study aim, specificity of the participant group and anticipated quality of focus group dialogue, given a topic of interest to participants and facilitation by an experienced interviewer. This number was also anticipated to maximise each participant’s opportunity to contribute, support rapport building between group members and was aligned with recommendations for the Nominal Group Technique [30], which was used in the second focus group in each series.

### Data collection

**Fig. 1:**
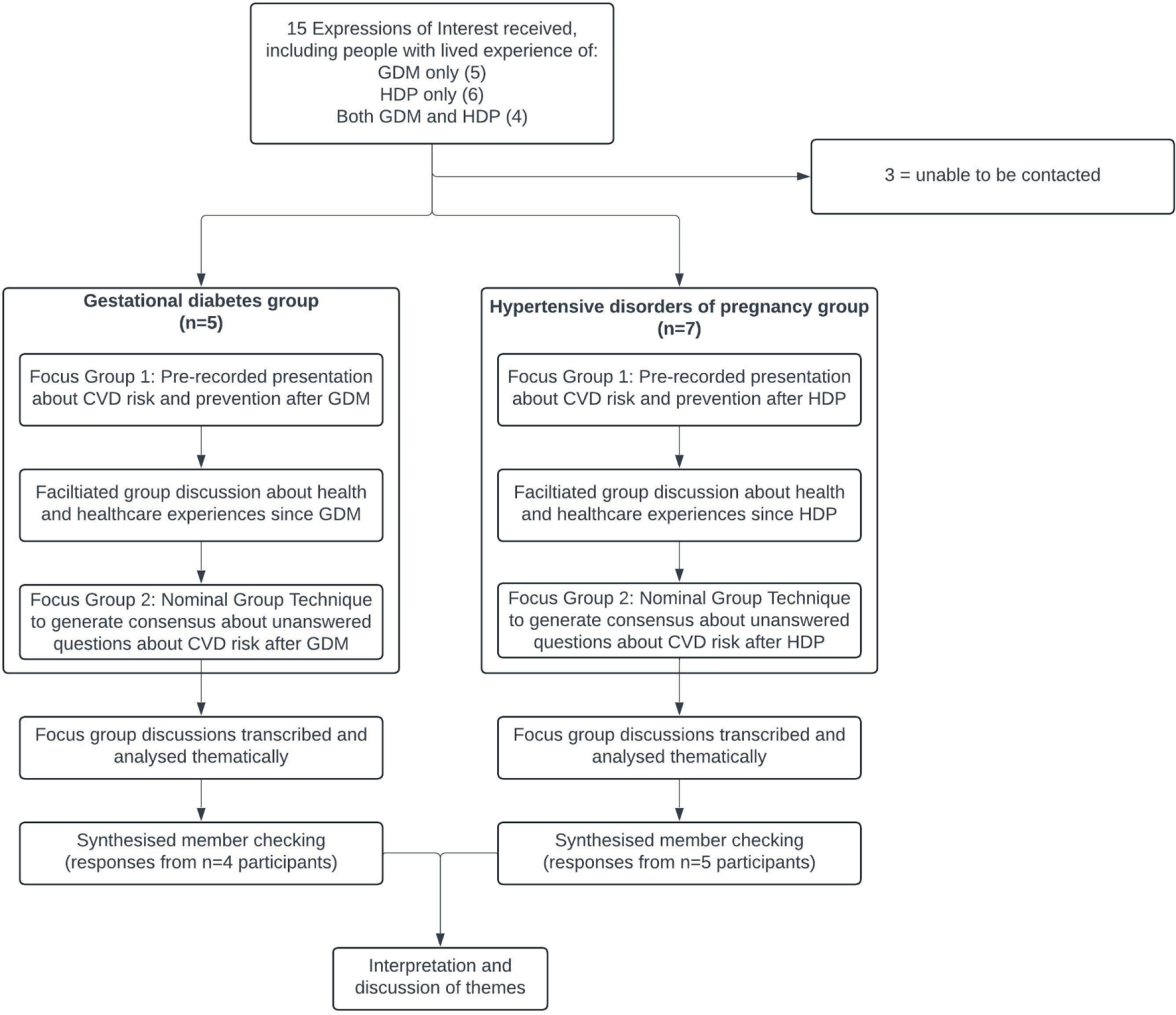
Recruitment and data collection process

Participants responded to a brief demographic survey and took part in a series of two focus groups, each two hours long, conducted via Zoom™ in February 2024. Participants were organised into focus groups depending on their pregnancy experience (see Fig. 1).

During the first focus group of each series, participants viewed a stimulus presentation that defined CVD, and it’s importance in women’s health, before presenting evidence about links between CVD and GDM [2] or HDP [3]. Participants then took part in a facilitated discussion about their health and healthcare experiences since their pregnancy (see Supplementary Material). The stimulus presentation was pre-recorded and delivered by a Professor of Life Course Epidemiology (GM) and a Clinical Professor and general practitioner (JD). The presentation combined background information about CVD and preventive health as well as statistical information, presented as icon arrays, about the increased risk of CVD after either GDM or HDP. Pre-recording the presentation allowed it to be scripted and reviewed by the LEE Group, who advised on accessibility, the use of plain English, and visual aspects in the presentation including the design of Powerpoint™ slides. It also meant the presenters did not attend the focus groups, helping to ensure that participants would feel free to offer their unfiltered reactions to it. Questions raised in the first focus group were collected for further discussion during the second focus group, held within one week.

**Fig. 2:**
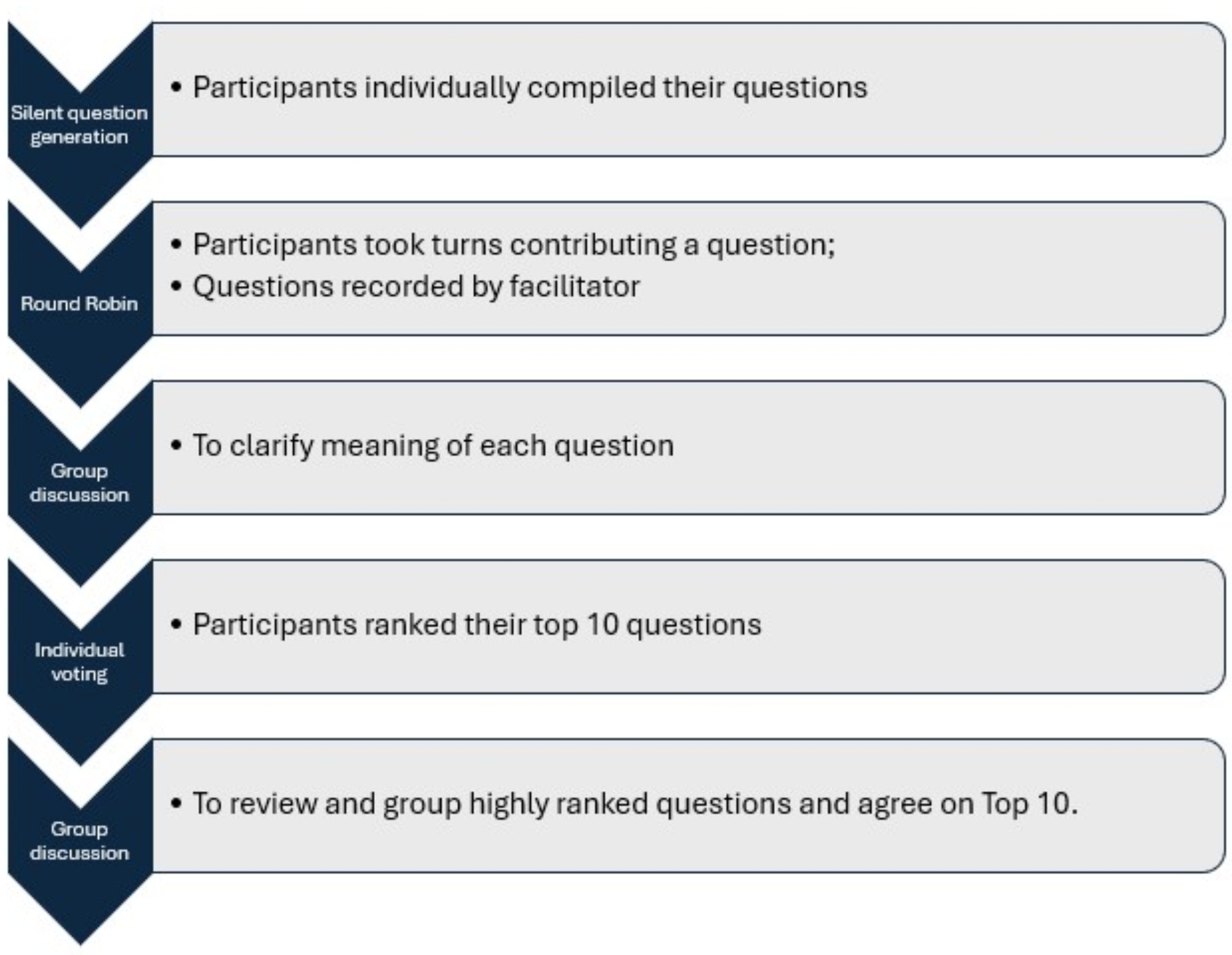
The phases of nominal group technique

In the second focus group, we used Nominal Group Technique (NGT) [30] to reach consensus about participants’ most important unanswered questions about the link between CVD and GDM or HDP. Nominal Group Technique [30, 31] is a structured approach to consensus building, that used phased individual reflection, group discussion and voting (see Fig. 2). In the ‘round robin’ phase, we used Jamboard™ (an online collaborative whiteboard) to collect participants’ questions following the presentation and discussion in the first focus group. Question clarification and grouping followed, and individual ranking using Slido™ polls and group discussion were used iteratively to reach an agreed top 10 questions.

Each focus group was co-facilitated by an experienced qualitative researcher with experience discussing sensitive topics in focus groups (BJ) and a member of the LEE Group (LH and VC, for the GDM and HDP groups respectively). Focus groups were scheduled at times that were accessible in all Australian time zones, and as preferred by participants (daytime for GDM; evening for HDP). Participants were welcome to have their children with them during focus group sessions, and to take breaks as needed. Each participant received a care package prior to their first focus group. Care packages contained a University of Queensland branded pen and notebook, and refreshments in the form of a cookie and herbal teas. Each participant also received an $80 gift card as a thank you after each session. Focus groups were digitally recorded, transcribed verbatim and anonymised using pseudonyms. Comments made via the chat function of Zoom™ were also collected and added to the transcript.

### Data analysis

Demographic data were analysed using descriptive statistics, with participant postcode used to classify their location according to the Monash Modified Model (MMM; [32]). Interview transcripts were analysed thematically [33] using in NVivo 14 [34]. The thematic analysis comprised five phases, delivering rich and nuanced understandings of the data. The first author read each transcript (Phase 1) and generated codes inductively (Phase 2), before discussing these with another author (JD) and the LEE Group to agree on preliminary themes (Phase 3). These themes were then further refined, defined and named (Phase 4), reviewing within and between themes and against the whole data set. This process continued iteratively and recursively, until each theme was clearly defined (Phase 5).

As part of Phase 5, we undertook Synthesised Member Checking (SMC) [35] to verify the trustworthiness of findings. We recontacted each participant via email approximately six months after their focus groups, with a brief message inviting them to review a plain English summary of the draft themes and provide feedback. Participant responses were incorporated into the data set and used to refine and finalise themes, which were then agreed upon by the research team and the LEE Group.

In this paper, quotes illustrating each theme are attributed to individuals using pseudonyms. Where needed for clarity and brevity, words have been inserted into quotes (denoted by [square brackets]) or omitted (denoted by…). Reported speech is indicated by inverted commas.

## Results

Fifteen people with lived experience of GDM, HDP or both were nominated by relevant health consumer organisations to take part in the study (Fig. 1). Upon follow up, five people agreed to take part in the GDM focus groups, seven agreed to take part in the HDP focus groups, and three were unable to be contacted. All participants identified as women (Table 1). Most participants were aged in their thirties and lived in either metropolitan (MMM1) or rural (MMM3-5) areas. No participants lived in regional (MMM2) or remote areas (MMM6-7). One participant identified as culturally and linguistically diverse and one identified as living with a chronic disease or disability. In the HDP group, four participants had been diagnosed with preeclampsia and three had experienced *H*aemolysis, *E*levated *L*iver enzymes, and *L*ow *P*latelets (HELLP) syndrome. HELLP is a serious HDP, and more severe form of preeclampsia. Participants had experienced their pregnancy complications between one and 15 years prior (median three years). At the Synthesised Member Checking stage, four of five GDM participants and five of seven HDP participants took part, and confirmed strong agreement with the draft themes.

**Table 1:** Participant demographics (*n* = 12)

Characteristic		HDP (*n* = 7)	GDM (*n* = 5)
		*n* (%)	*n* (%)
Age	18–29 years	2 (29)	-
	30–39 years	3 (43)	5 (100)
	40–49 years	2 (29)	-
Location	Metropolitan city (MMM1)	2 (29)	4 (80)
	Large rural town (MMM3)	2 (29)	-
	Medium rural town (MMM4)	1 (14)	-
	Small rural town (MMM5)	2 (29)	1 (20)
Educational attainment	Vocational or trade qualification	6 (86)	-
	Bachelor’s degree	-	5 (100)
	Postgraduate degree	1 (14)	-
Gravida	1 pregnancy	1 (14)	3 (60)
	2 pregnancies	3 (43)	1 (20)
	3 pregnancies	1 (14)	1 (20)
	4 + pregnancies	2 (29)	-
Parity	1 birth	2 (29)	3 (60)
	2 births	4 (57)	2 (40)
	3 births	-	-
	4 + births	1 (14)	-

During focus group discussions, participants indicated that they were interested in, but previously unaware of any links between their pregnancy complication and their future risk of CVD. Distinct themes were generated from the discussions in the GDM and HDP focus groups. From the GDM focus groups, three themes captured participants’ health and healthcare experiences since their GDM: ‘*A distressing diagnosis*’, ‘*Degrees of diabetes’*, and *‘Balancing motherhood and self-care’*, while two themes captured HDP focus group discussions: ‘*Women’s concerns were dismissed’*, and ‘*Wanting follow up at the right time and with the right person’*.

### Themes generated from GDM focus group discussions

#### ‘A distressing diagnosis’

Women’s accounts of being diagnosed with GDM emphasised that it had been a distressing and isolating experience. Some regarded diabetes as being linked to “poor health, poor life choices in terms of food and exercise” (Laura), pointing to an individualised sense of responsibility for their GDM. Immediately following their diagnosis, women had felt “crammed full of all this information” (Laura), which they sometimes perceived as “traumatic” and “full on” (Laura).

*I was already terrified. And then “oh now you’ve got this thing that if you don’t eat right or if you don’t exercise properly*,* it’s your fault if something happens to your baby” like I just think the way it’s delivered is really*,* it can be quite damaging. (Amy)*

Participants perceived that, after their GDM diagnosis, their maternity care had shrunk to “always all about baby and what it’s doing to baby,” (Amy) without much consideration of their own wellbeing or experience.

*Before we discussed diet*,* before we’ve discussed impacts*,* it was “Your baby could be stillborn”. And that knowledge*,* when you’re a first-time mum and especially I’ve had early pregnancy losses as well to have that thrown in the mix as… There was a lot to unpack. (Laura)*

*Once I had had my son everything kind of went out the window. They kind of didn’t even remember I had gestational diabetes. They were giving me whatever meals… in the hospital. (Rebecca)*

While some participants had positive interactions with healthcare professionals, for others, efforts to make dietary changes had unintended and unwelcome social impacts.

*I was really fortunate to have a good diabetic educator. She didn’t start the conversation with those horrible things. Yeah*,* she also made it super clear that it wasn’t my fault… it’s not because you’ve eaten too much of this or too much of that. It happens with really healthy people too. (Rebecca)*

*I have no guilt in it [diet*,* before GDM] and that sort of shifting perspective of how I approached food was very challenging for me throughout my pregnancy. I did manage to control all my sugars with diet alone… but it was so sad… Most of my social interactions are around food… which is very difficult for me personally. (Maria)*

For at least one participant, their experiences around GDM had prompted them to disengage from other aspects of healthcare which they perceived to be judgemental.

*[Baby] hasn’t been to a single maternal child health appointment because… I don’t feel like feeling like a bad mum or like I’m not doing the right thing. (Amy)*

#### ‘Degrees of diabetes’

Women’s accounts of their GDM indicated some scepticism about their diagnosis.

*I came back as like borderline. So*,* it was like 0.1 or something off what it like I could have been not gestational diabetes. (Rebecca)*

This was at least partly driven by awareness of variation in the way that GDM was defined, both within Australia and internationally, as well as changes to screening approaches over time.

*I don’t have it. This is a lie!… I’m from [overseas]*,* the numbers*,* for diagnosis are completely different*,* so a lot of my friends were like “oh she should be fine. Like that’s nothing”. (Maria)*

*If you’ve gone to X hospital or it was two or three years ago… So those cut offs… and the change between those cut offs*,* just between hospitals in the same city*,* is definitely something worth considering. (Laura)*

While participants were generally aware of their increased risk of developing T2DM subsequent to GDM, none were aware of a link with future CVD. Participants’ awareness of the variation in the screening and diagnosis of GDM led them to question how they should interpret information about their future CVD risk, and whether the link between GDM and CVD would apply to them.

*The two tests between my two pregnancies… were really different. [It’s] not that I think I didn’t have GD [gestational diabetes]*,* but… if the testing is different and it’s different depending on which hospital you go to*,* how are you supposed to understand… what your risk factors are? (Amy)*

*It just cannot be reasonable to say that somebody who’s been diagnosed with GDM but fully controlled their blood sugars*,* has got the same risk factors [for future CVD] as somebody who*,* try all they might*,* was on the highest dose of insulin and they did everything they could possibly do*,* but unfortunately their body was doing what it was doing. (Jennifer)*

#### ‘Balancing motherhood and self-care’

While participants were generally interested to know more about the relationship between GDM and future CVD, they were cautious about the usefulness of this information to new mothers, especially after traumatic birth experiences.

*First and foremost*,* you can tell me that I need to eat well*,* and I need to exercise. But I have a new baby and a toddler. And I don’t have time for that. (Amy)*

*I have also had a traumatic birth… So*,* I’m actually mentally not coping as well as I could be and because of that I’m not taking as good care of myself as I should be. Hence the diet is not right. There’s no time for me to exercise and all of that. So*,* I feel like yes*,* it’s important… but there’s a lot going on. (Rebecca)*

The life stage of intensive mothering had continued for participants for at least a couple of years, and in some cases until their children had started school.

*My eldest is five and my youngest is two and I am now thinking maybe I might be able to carve out… one exercise session for just me*,* a week. (Amy; Participant’s emphasis)*

*She’s [child] now starting at Kindy… prior to this point… there was like zero times for anything… But this year is all about me*,*… I’m now for the first time*,* really able to set some time aside [for exercise]… only one session a week. (Jennifer)*

Participants were aware of and followed recommendations for annual monitoring over their diabetes status, but beyond that, most perceived that knowing about increased risk of future CVD was unlikely to influence them.

It’s just going to add guilt. It’s not going to change anything. (Maria)

*So even though I say mention it [CVD risk]*,* and it would have been nice to know… It wouldn’t have changed a single thing… You are just thinking about baby. You’re not thinking about your prospective future health. (Jennifer)*

Nonetheless, if such information was to be shared, the consensus was that it was best done in the context of an ongoing therapeutic relationship.

*[Telling your story to] a new person every time is… horrible… They can’t deliver a message that I want to hear. (Amy)*

### Themes generated from HDP focus group discussions

#### ‘Women’s concerns were dismissed’

Women’s experiences of healthcare during and after their HDP were characterised by feeling “fobbed off” (Stephanie). During pregnancy, they perceived that their symptoms and concerns were dismissed.

*I was a hysterical first-time mother… They thought there was nothing wrong*,* and that I was just having an anxiety attack. I wasn’t. It was my liver failing. (Stephanie)*

This meant that some women felt responsible for maintaining a record of what had happened during their pregnancy, to inform their subsequent health care providers and their own decision making.

*They [maternity care providers] never once wrote in my book [pregnancy health record]. Ever.… I was the one that was typing everything into… a note in my phone… So*,* if I do decide on having a third [baby] one day*,* I’ve actually got the information. (Melissa)*

Some women felt that their health and wellbeing had been overly de-prioritised in favour of prolonging the pregnancy for the benefit of their baby.

*They literally waited until the eleventh hour [to] do my delivery [at 36 weeks]… It feels like they just wait too long. I know they’re weighing up the difference of gestation*,* like getting a baby further… [but] why is it this be all*,* end all of just keeping a baby cooking? (Jessica)*

Postnatally, participants described very little attention being paid to their hypertension, either in the first few weeks after birth or in the years since.

*“You can now go back and see your GP and finish off weaning off Labetalol”. That was literally the only conversation. Everything else was… “oh the [caesarean incision] site looks good. They did fantastic stitching.” It was very*,* very trivial… There wasn’t even a conversation about future pregnancies. (Jessica)*

*No one’s ever mentioned it [HDP] to me again since… There’s been no nothing. (Diana)*

*Nobody asks about pregnancy… It’s like a whole secret has happened… Like nobody checks on you*,* really at that six-week mark. Nobody really checks on you a year later to see how you’re going. It’s just really forgotten time. (Melissa)*

Even when participants had tried to seek follow up care specifically regarding their HDP, they had been unsuccessful.

*I felt the GPs [General Practitioners]*,* nobody asks you what your previous history is. When you say I’ve had preeclampsia*,* they just brush it aside… you need someone who knows and has the experience*,* and I just don’t think GPs*,* whether they’re not being taught this or they’re not seeing it enough. (Melissa)*

This lack of follow up care was particularly challenging for participants who linked ongoing health issues to their HDP. In some cases, participants valued the follow up care that their child’s prematurity continued to attract but noted the contrast with the support offered to them.

I never actually fully recovered but they fobbed everything off like it’s nothing. (Stephanie)

She’s little. I’ve still got follow-ups for her, but there’s nothing for me. (Theresa)

#### ‘Wanting follow up at the right time and with the right person’

Although the link between HDP and future CVD risk was new information to participants, they were interested in knowing more, as they perceived it would support them to making informed decision about their health and healthcare.

*This is the first time I’ve ever heard any of this. It was literally “Congratulations*,* you had a baby*,* see ya.” And because my blood pressure did go back to normal afterwards*,* there was no mention of anything after. So*,* I’m really glad to hear this*,* actually. (Diana)*

*It’s informed consent where you’re aware of something and you know the risk*,* and… you’re making decisions and choices. (Jessica)*

Participants wanted improved follow up with health care professionals. However, they emphasised that follow up should be streamlined and routine, and with a healthcare professional knowledgeable and able to provide sound advice.

*If we got the reminder message… saying… go to your GP and… they don’t know anything about it or… dismiss it*,* then you’re just back to… [It needs to be] something from someone who’s actually trained in this and not just your GP*,* that might not know what they’re talking about. (Diana)*

Nonetheless, most participants also reported that they would not have had capacity to think about their own long-term health during the first few months after HDP, while adjusting to motherhood and recovering physically and emotionally from a complicated pregnancy and birth.

*At six weeks I was just a crying mess and had only just come out of the special care nursery with my baby… I think it would have definitely bombarded me at that time. (Diana)*

*I feel like about 3 months would have been good for me because… I’ve settled in a bit. I’ve wrapped my head around everything that’s happened. I’m physically recovered. (Melissa)*

*I’d say even around like the four to six month vaccinations would be a decent time for the doctor to pull you aside. “OK*,* well*,* while you’re here*,* let’s discuss these risk factors”. (Emma)*

### Unanswered questions

The unanswered questions prioritised by each group are presented in Table 2. These questions fell into three broad categories: Improving maternity care, preventing CVD and (for women with a history of HDP), concerns beyond HDP.

**Table 2:** Unanswered questions prioritised by people with lived experience

	Hypertensive disorders of pregnancy	Gestational diabetes
Improving maternity care	• What is the role of social support during a complicated pregnancy? • What are women’s trauma-informed postnatal care and social support needs in the first several weeks after birth, following HDP? • Better detection and treatment options for postnatal pre-eclampsia.	• How can we re-balance the conversation during pregnancy to include the woman’s wellbeing not just the baby? • Why is there so much variation in approaches to screening, diagnosis and management of GDM? Do these different approaches improve women’s outcomes? • Given the variation in approaches to screening for and diagnosing GDM, how should women interpret risk information both during and after pregnancy? • What are normal blood sugar levels during pregnancy? How do we know?
Preventing CVD	• What and when would specialist follow up be beneficial to women after a pregnancy affected by HDP? • Would other medications (e.g. low doses of BP medications) might be useful after HDP to prevent CVD? When would these be useful? • What are the education needs of GPs about CVD and HDP, and how often are women asked about their obstetric history in primary care? • If you have a family history of pre-eclampsia, are there effective ways of preventing HDP *before* pregnancy (i.e. earlier in life)? • What moderates and mitigates the link between CVD and HDP? e.g. timing and severity of HDP; presence of multiple complications, genetic factors, family history. • How is the risk of CVD following HDP managed in different contexts, and with what outcomes and experiences for women?	• Amongst all the risk factors for CVD, how important is GDM? • Will knowing about the link between GDM and CVD help improve women’s health or just create extra stress? • What is the role of other factors (e.g. family history, maternal stress) in the link between GDM and CVD? • Is there a link between interventions during pregnancy, labour and birth, and increased risk of CVD? • If GDM is specific to pregnancy, what is the mechanism by which it could affect long term health? • Does the severity of your GDM and its management during and after pregnancy, alter the risk of CVD in the future?
Beyond CVD	• Are there other chronic health issues that women with a history of HDP are at increased risk of? e.g. vascular dementia, clots, POTS, DVTs.	

## Discussion

This study set out to describe women’s preferences and experiences of preventive healthcare in the years after GDM or HDP, and to identify their unanswered questions about the association between their pregnancy complication and future CVD risk. Although ongoing monitoring of women with a history of GDM is more embedded in routine care [13], neither group indicated they had been aware of their increased CVD risk prior to this study. This suggests that information about CVD risk may not be consistently or effectively shared with women after a pregnancy affected by GDM or HDP. Although GDM and HDP share several risk factors, there were important differences in the experiences reported by women in this study. These differences in the experience of being diagnosed with GDM compared to HDP have implications for CVD preventive healthcare.

Beyond the stress evident in both participant groups’ experiences, participants in the GDM group described the experience as distressing. The concept of diabetes distress encapsulates “the worries, concerns, fears and threats that are associated with struggling with a demanding chronic disease like diabetes over time, including its management, threats of complications, potential loss of functioning and concerns about access to care”([36], p803). It is distinct from depression and is regarded as a normal emotional response to being diagnosed with a complex chronic disease. Diabetes distress is receiving increasing scholarly attention, but mostly in the context of Type 1 and Type 2 diabetes [36], rather than gestational diabetes.

For participants with lived experience of GDM, their distress may have stemmed from the perceived stigma and judgement associated with the diagnosis. While stigma in association with GDM has been reported previously [37–39], our findings add to this by suggesting that participants may respond to stigma by seeking to distance themselves from their diagnosis. This in turn may complicate efforts to provide CVD preventive healthcare after GDM, as doing so revisits the stigma and distress of their pregnancy complication. Routine assessment of diabetes distress during a GDM affected pregnancy, efforts to destigmatise GDM diagnosis, and appropriate psychosocial support both during and after pregnancy, may improve both women’s immediate pregnancy experiences and outcomes, as well as their long-term engagement with preventive healthcare and health promoting behaviours [40, 41]. The inclusion of psychosocial support in postnatal GDM interventions has thus far been rare [41], and should be the focus of future research.

Similar to participants in other qualitative studies [42], both groups in this study perceived that their pregnancy complication had narrowed the focus of their maternity care to the health of their baby, to the exclusion of their own wellbeing. Maintaining a woman-centred approach during a complicated pregnancy is challenging, at least partly because of fragmentation of care that may increase the chances of women ‘falling through the gaps’ [43]. Other studies have also found that women’s involvement in shared decision making may be constrained during a complicated pregnancy [44], which in turn is also associated with traumatic birth experiences [45]. This constellation of circumstances ’lack of woman-centred care and involvement in decision-making, fragmentation of care, and increased potential for traumatic birth experiences ’may explain the findings of other studies that women need time to recover psychologically after HDP before considering health optimisation [16, 42, 46]. Each of those circumstances is also amenable to improvement; interventions which improve women’s experiences of care during and soon after a complicated pregnancy should be included in future research, including their impact on women’s capacity to take up preventive healthcare sooner.

Beyond their postnatal maternity care, and especially after HDP, women appear to have encountered little ongoing follow up of their health. The transition from hospital-based maternity care to community-based care is also known to be vulnerable to fragmentation and missed communication [5, 25, 42, 47–49]. There has been sustained effort in Australia to establish routine T2DM screening after GDM [50]. For HDP, clinical practice guidelines are available in Australia [22, 23] and internationally for long-term healthcare, but have not been widely implemented [51]. Primary healthcare professionals may also lack knowledge about links between pregnancy complications and future CVD risk or be reluctant to discuss this with women [49]. Both the content of risk information, and the manner in which it is communicated are important contributors to women’s uptake of preventive health care [52]. It is vital that efforts to encourage women to seek preventive health advice are preceded by further research with primary care providers to establish feasible clinical pathways and ensure primary care providers have the necessary knowledge and information to support patient needs. This was also a priority question for participants in the HDP group.

On the whole, participants in this study were previously unaware of the association between their pregnancy complication and increased risk of future CVD and wanted to know more. Women who are aware of their increased risk of CVD may be more likely to seek preventive healthcare to manage that risk, such as regular blood pressure and lipid level monitoring [53], potentially creating more long term opportunities to support health optimisation and respond more quickly to emerging clinical indications. However, both participant groups prioritised questions seeking more nuance in information about their increased risk of CVD, for example to understand the role of timing of onset, severity of symptoms of their pregnancy complication. Especially in the case of participants with lived experience of GDM, efforts to reduce unnecessary variation in the screening and diagnosis of GDM could support greater understanding of their CVD risks in the months and years afterwards.

The years after childbirth are also recognised as a time when women’s own healthcare needs may be eclipsed by the needs of their children [46, 54], a situation which may persist until midlife [55]. Beyond awareness raising, both groups emphasised the challenges of prioritising their own health when mothering young children. Other studies have pointed to time poverty and time intensity, which often accompany motherhood, as social determinants of health [56]. Current approaches to CVD prevention after pregnancy complications mostly target lifestyle change, but sustainable change has proven difficult to achieve, with the main barrier cited being time, often borne out in poor recruitment and retention rates [16, 17]. An overemphasis on individual lifestyle intervention, especially without appropriate support and attention to the broader social determinants of women’s health, may create unintended harm [57]. Strategies such as accessible high quality childcare and patient navigators, may support women to carve out space for their own health and wellbeing, separate from their role as mothers [14], and should be considered in future studies.

Strengths and limitations.

Although this was a small qualitative study, the authentic involvement of the Lived Experience Expert Group in all aspects of the project was a great strength. The inclusion of Synthesised Member Checking (involving both participants and the lived experience expert group) strengthened confidence in our findings. Nonetheless, we recognise that we recruited women who had chosen to connect with and remain connected with peer support networks, perhaps because of their own atypical experiences. It was more challenging to recruit women with lived experience of GDM, than HDP, perhaps reflecting the absence of a maternity consumer organisation focussed on GDM, and women’s reluctance to revisit a distressing and stigmatising experience. We also acknowledge that while the virtual mode allowed us to include participants from across Australia, it may also have excluded those with lower digital literacy. This was partly mitigated by the offer of digital familiarisation sessions before each focus group; however, no participants accessed this support suggesting that information about the study may not have reached those with lower digital literacy, or they may have self-excluded. We were also only able to conduct the focus groups in English; the needs and experiences of non-English speaking women warrant further research. Finally, participants were also at various points after their pregnancy complication experience (up to 15 years). While this did enable participants to reflect on their healthcare experiences since, it also may have introduced recall bias. We also recognise that health services offered to women after GDM and HDP may have changed since participants experiences, however our focus was on their CVD follow up care more so than on their maternity care.

## Conclusion

Our findings indicate that women’s capacity to engage in preventive health in the years after a pregnancy impacted by GDM and HDP may be attenuated by their maternity care experiences and the accessibility of suitable primary care pathways. In conceptualising future research on CVD prevention after GDP and HPD, consideration should be given to improving woman-centredness of maternity care, ensuring seamless transition back to primary care after a complicated pregnancy, and greater attention to the social determinants of health for new mothers. Future research should attend to the questions prioritised by participants in this study and involve people with lived experience in the codesign of interventions.

## Electronic supplementary material

Below is the link to the electronic supplementary material.

Supplementary Material 1

Supplementary Material 2

## Data Availability

Owing to the confidentiality of the information, the datasets analysed in this study are not publicly available. Upon reasonable request, they can be made accessible through the corresponding author.

## Abbreviations

CVD: Cardiovascular diseases
GDM: Gestational diabetes mellitus
GP: General Practitioner
GRIPP2: Guidance for Reporting Involvement of Patients and the Public
HDP: Hypertensive disorders of pregnancy
LEE: Group Lived Experience Expert Group
NGT: Nominal Group Technique
SMC: Synthesised Member Checking
