# Supplementary material for: Women’s health and healthcare experiences in the years after gestational diabetes or hypertensive disorders of pregnancy
Source: BMC Pregnancy Childbirth. 2025 Feb 14;25:158. doi: 10.1186/s12884-025-07296-7 (PMC11827438; doi:10.1186/s12884-025-07296-7)
Supplement: Supplementary file 1 — Supplementary Material 1 [file 12884_2025_7296_MOESM1_ESM.docx]

**Women’s Health After Pregnancy Complications and Loss**

**(WHAPCAL)**

**Guide for Workshop Series**

**Day One – Discussing the evidence (a Tuesday)**

| Preworkshop | - Optional zoom familiarisation session |
| --- | --- |
| Mins 0-30 | - Welcome, housekeeping, introductions/icebreaker:   - Please tell us your name, what network you represent, and a “catchphrase” you are known for?   - Group agreement |
| Mins 30-45 | - Pre-recorded presentation/s from Prof Gita Mishra and Prof Jenny Doust |
| Mins 45-105 | - Facilitated discussion   - What would you like researchers to understand about the months and years after [specific PCL experience]?   - Tell me about your interactions with health care professionals in the months and years after [specific PCL experience].   - Did you talk to them, or anyone else, about your future health? Would you have wanted to?   - When might be the best time to open a conversation with a woman after a [specific PCL experience] about her future health?   - How would you like to receive information about that? (eg in person consult; text message directing to website etc)   - What might work for women with [specific PCL experience]? |
| Mins 105-120 | - Workshop wrap up, brief centring activity |

**Day Two – Nominal Group (Thursday of the same week)**

| Mins 0-15 | - Check in and reconfirm Group Agreement |
| --- | --- |
| Mins 15-20 | - Silent ideas generation: “Thinking back on the two presentations on Tuesday, and all of our conversation, what are your unanswered questions about possible links between [specific PCL experience] and future CVD risk?” |
| Mins 20-40 | - Round robin: Each participant will be invited to share a single idea, continuing in turn until no new ideas are offered. Ideas will be recorded on a shared screen, verbatim, without comment or discussion |
| Mins 40-70 | - Clarification/Grouping: Facilitated discussion to clarify, elaborate, nest or debate (but not eliminate) ideas. |
| Mins 80-85 | - Participants undertake an initial voting round to identify their top ten priorities. - Results (mean score for each priority) will be immediately shared with participants. |
| Mins 85-105 | - In breakout rooms, pairs will discuss priorities whose mean score placed them in the group’s top 20 and create a rank ordered list. - Pairs’ rank ordered lists will be collated and mean scores reported back to the group for each priority. |
| Mins 105-120 | - Discussion will follow to generate consensus on the top 10 ideas. |
| Mins 120-135 | Workshop close out   - Check in with each member: Take home message for the research team? - Brief centring activity |
